# Generation and Characterization of Human-Mouse STING Chimeras That Allow DENV Replication in Mouse Cells

**DOI:** 10.1128/msphere.00914-21

**Published:** 2022-04-28

**Authors:** Tongtong Zhu, Laurence G. Webb, Jeury Veloz, Maris Wilkins, Sebastian Aguirre, Ana Fernandez-Sesma

**Affiliations:** a Department of Microbiology, Icahn School of Medicine at Mount Sinai, New York, New York, USA; b Graduate School of Biomedical Sciences, Icahn School of Medicine at Mount Sinai, New York, New York, USA; University of Michigan-Ann Arbor

**Keywords:** dengue, flavivirus, innate immunity, recombinant proteins, viral antagonism

## Abstract

Our group was the first to describe direct antagonism of the cyclic GMP-AMP synthase (cGAS)/stimulator of interferon genes (STING) pathway by dengue virus (DENV) in human cells, and here, we report new findings on the characterization of the interaction between the DENV nonstructural protein 2B (NS2B)-NS3 (NS2B3) protease complex and STING. We demonstrate interactions between NS2B and the transmembrane domains of human STING and between NS3 and a portion of the cytoplasmic C-terminal domain of human STING. One significant obstacle we face today in the DENV field is the lack of small animal models available that can effectively recapitulate DENV pathogenesis in the early events of infection. The existing mouse models are either immunocompromised mice lacking interferon (IFN) receptors or “humanized” mice reconstituted with human stem cells. However, both approaches fail to capture important aspects of human pathogenesis because they lack critical innate immunity components or have deficiencies in immune cell development or maintenance. As an important step toward developing an immunocompetent mouse model for DENV, we have generated two chimeric human-mouse STING constructs that have promise in retaining both cleavability by NS2B3 and signaling capacity in the mouse.

**IMPORTANCE** This article characterizes the interaction between human STING and DENV viral protease complex NS2B3 by constructing serial deletion mutants of STING. Our findings suggest that DENV nonstructural protein NS2B interacts with the transmembrane domains and NS3 with the C-terminal cyclic dinucleotide binding domain of human STING. Furthermore, as there exists no ideal immunocompetent murine model that can simultaneously support robust DENV replication and recapitulate the clinical manifestation of dengue disease observed in humans, we expressed and characterized two promising human-mouse chimeric STING constructs that can be used for developing a relevant transgenic mouse model to study dengue in the future. Both constructs can activate normal IFN responses in the overexpression system and be cleaved under infection conditions. We believe our findings offer a roadmap to the further development of a murine model that can greatly facilitate antiviral discoveries and vaccine research for DENV.

## INTRODUCTION

Dengue virus (DENV) is the most prevalent arthropod-borne virus in the world (3). Out of the estimated 400 million infections that occur every year, around a quarter present as nonspecific febrile illness (also called dengue fever), and a small percentage of cases will develop into more severe forms of hemorrhagic fever or circulatory failure (4). DENV circulates in human populations in the form of four serotypes (DENV1 to -4), and any previous exposure to one of the four serotypes can lead to a more clinically severe form of infection by another serotype (5–7). Even when mortality from these severe forms of dengue is low in countries that have robust health infrastructures, the heavy and growing social-economic burdens on health providers in regions of endemicity continue to pose a significant global health challenge (8).

DENV is the first flavivirus found to have antagonistic activity against the cyclic GMP-AMP (cGAMP) synthase (cGAS)/stimulator of interferon genes (STING) pathway, opening the field of RNA viruses to new directions in innate immune antagonism (9). This important cytosolic DNA-sensing pathway has been demonstrated to be implicated in many different flavivirus infections of mammalian systems (10, 11). In the context of DENV infection, cGAS has been shown to be degraded during DENV infection through an autophagy-lysosome-dependent mechanism (12). Along with the discovery that human STING (hSTING) is the target for proteolytic degradation by the viral nonstructural protein 2B (NS2B)-NS3 (NS2B3) protease complex during infection, we now know that DENV antagonizes both components of this important innate immune sensing pathway during replication (13, 14). Given the additional observations that murine STING (mSTING) is not susceptible to cleavage by the DENV protease while human STING can be efficiently cleaved, it appears that DENV is well adapted to replicate in human but not murine cells (13–16).

Over the years, there have been a few publications reporting on the putative cleavage site of hSTING by DENV NS2B3, but none have proven to be conclusive (13, 14, 17, 18). The most likely cleavage site for hSTING is proposed to be between arginine-78 and glycine-79, as examined by Stabell et al., and this is in line with the proposed preferred substrate pattern for DENV NS2B3 protease (17, 19). This could explain why mSTING is resistant to cleavage, as the corresponding amino acids at this location for the murine version are glutamine and glycine. Nevertheless, having no direct evidence to prove cleavage occurs at this location, the authors did not rule out this sequence as an important protease binding determinant instead. More studies are needed to characterize both binding and cleaving determinants between NS2B3 and human STING.

Many attempts have been made to propagate DENV and recapitulate dengue disease in animal models (20). Of particular interest to our investigation, the development of an immunocompetent murine model to study DENV pathogenesis has eluded researchers for years. Many reported mouse models either use immunocompetent mice that need to be infected with a high multiplicity of infection (MOI) of DENV or immunocompromised and immunodeficient mice that express human immune system components (1, 2, 21). Nevertheless, none of the models can fully support a high viremia and at the same time capture the disease manifestations of dengue infections in humans. We believe that a better mouse model can be constructed by generating a human-mouse chimeric STING construct that can both signal normally in a murine environment and be antagonized by DENV during infection. Towards that goal, we present here two recombinant STING proteins that exhibit robust signaling power and can support DENV replication when expressed in STING knockout (STING-KO) murine cells.

## RESULTS

### NS2B3 proteases from different DENV serotypes, but not from other flaviviruses, cleave hSTING.

**FIG 1:**
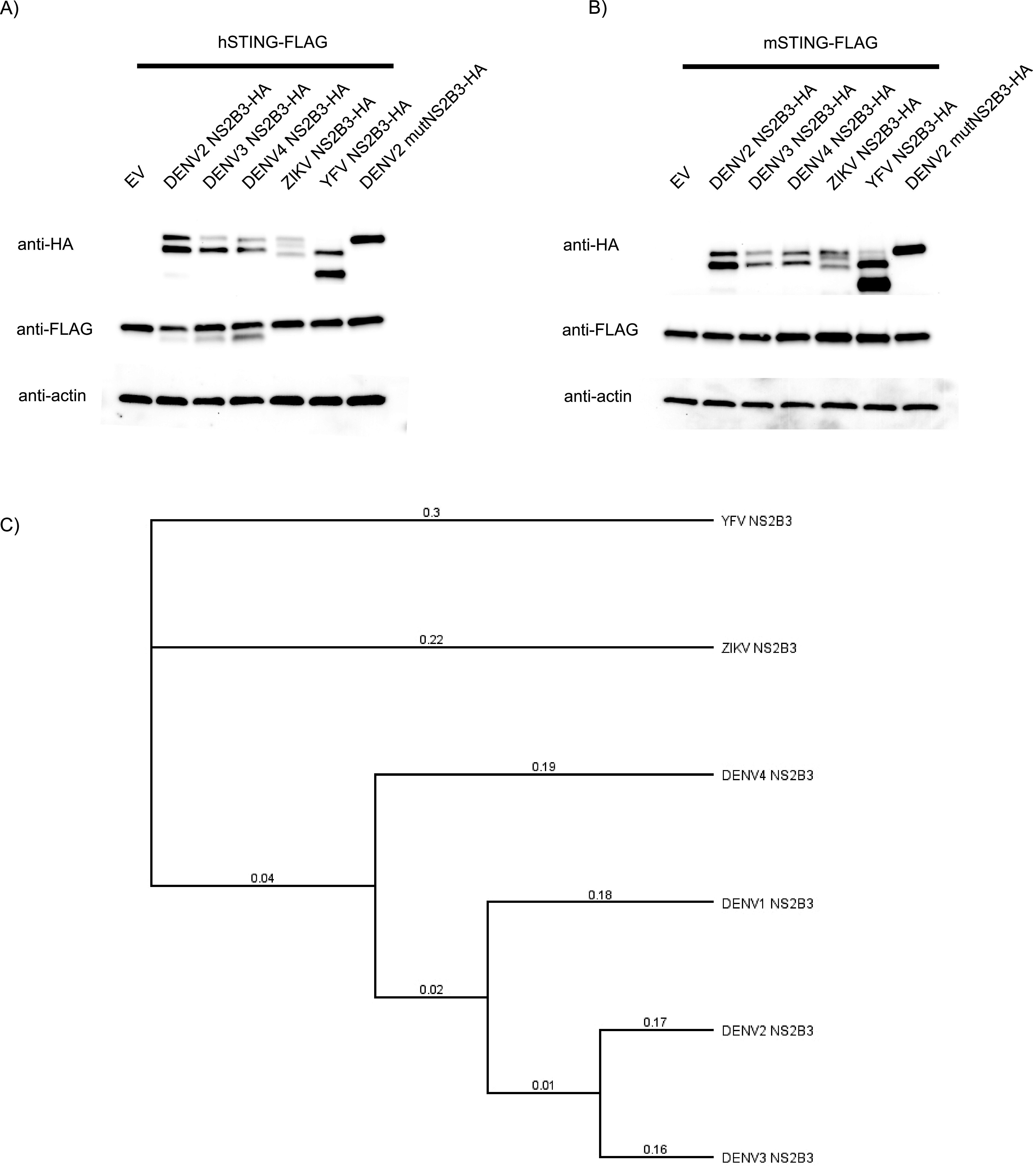
Testing hSTING and mSTING’s susceptibility to cleaving by different DENV protease complexes. (A) NS2B3 proteases from different DENV serotypes, but not other flaviviruses, cleave human STING (hSTING). Western blots of total cell lysates collected at 24 h post-cotransfection of hSTING-FLAG with empty vector and various NS2B3-HA constructs. mutNS2B3 has a replacement of S with A at position 135. (B) NS2B3 proteases from different DENV serotypes and other flaviviruses cannot cleave mouse STING (mSTING). Western blots of total cell lysates collected at 24 h post-cotransfection of mSTING-FLAG with empty vector and various NS2B3-HA constructs. (C) Phylogenetic tree constructed using amino acid sequences of DENV, YFV, and ZIKV NS2B3 proteases. The Geneious Aligner algorithm (Geneious Prime 2021.2.2) using the neighbor-joining method based on the Tamura-Nei genetic distance model was used to align multiple protein sequences and construct this phylogenetic tree. Substitutions per site are shown.

Previously, our laboratory (as well as the Yu group) reported that the DENV2 protease complex NS2B3 could interact with and cleave hSTING but not mSTING (13, 14). In those experiments, only NS2B3 of DENV2 strains 16681 (14) and New Guinea-C (13) were used. For this study, we tested the ability of the proteases from different DENV serotypes (DENV2 16681 strain, DENV3 Puerto Rico 2001 strain, and DENV4 Indonesia 1976 strain) to cleave hSTING. Using an overexpression system in HEK293T cells, we cotransfected NS2B3 of each of the DENV serotypes with hSTING (Fig. 1A) and mSTING (Fig. 1B). We also included the protease complexes of two other flaviviruses, Zika virus (ZIKV) French Polynesia strain and yellow fever virus (YFV) 17D strain (Fig. 1C), along with a catalytically inactive form of DENV2 NS2B3. Out of all the well-expressed experimental conditions, we saw that only the DENV NS2B3 proteases could degrade hSTING (Fig. 1A), while mSTING remained resistant to cleaving under all conditions (Fig. 1B). Interestingly, DENV4 NS2B3 seemed to cleave hSTING more efficiently than DENV2 NS2B3. This could potentially be yet another difference between DENV2 and DENV4, in addition to replication kinetics and the ability to induce the type I interferon (IFN-I) pathway during infection, as shown in our earlier report (Fig. S1 in the supplemental material) (22). Finally, we detected no cleavage of hSTING by ZIKV NS2B3, contrary to the observation in another report (18). However, the different outcome could be because a different strain of ZIKV was used in our experiments, further highlighting the differential nature of antagonism across viral species and strains.

### N-terminal cytoplasmic loop of hSTING is important for its cleavage by NS2B3.

**FIG 2:**
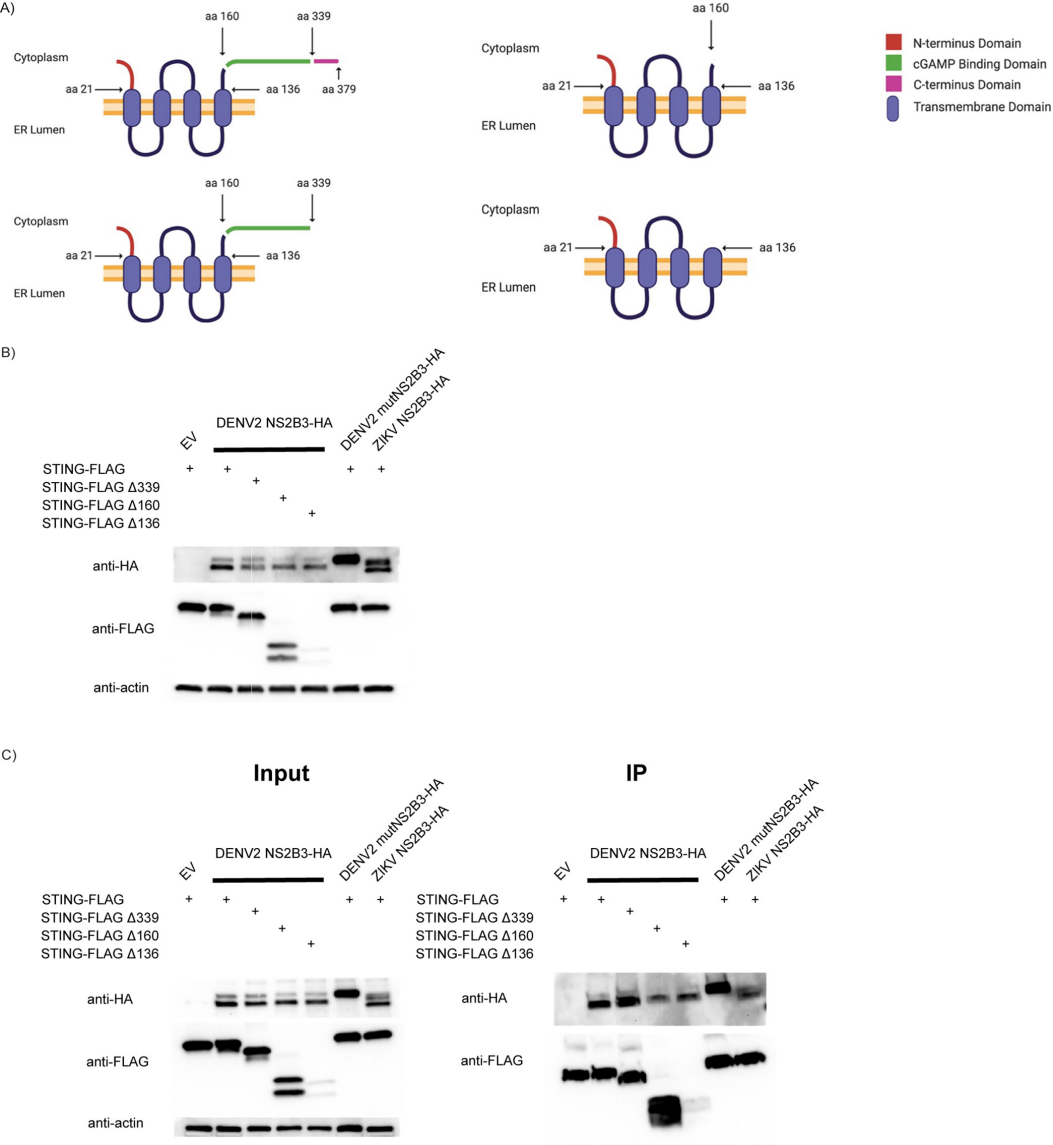

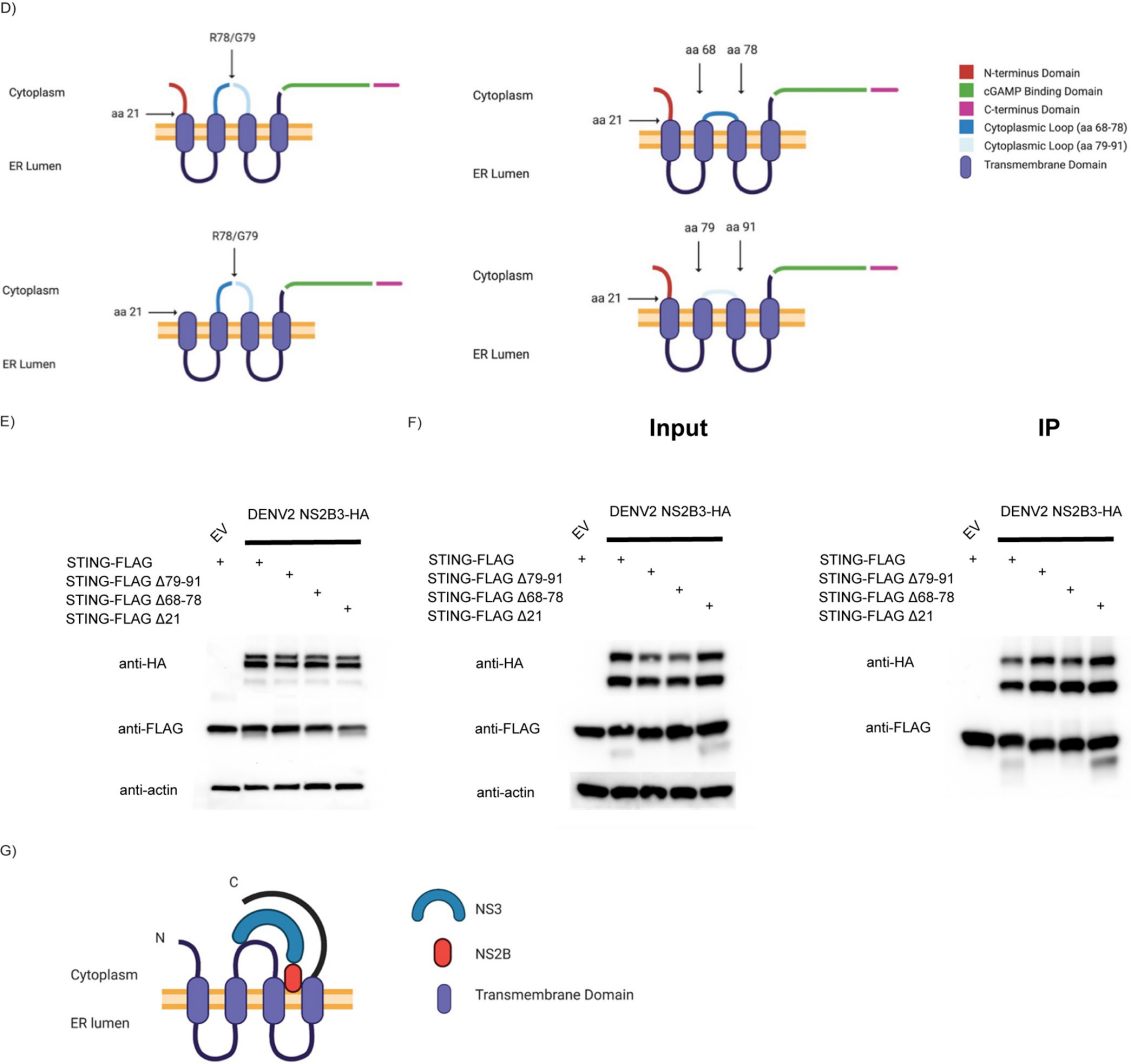
Mapping protein-protein interaction between human STING (hSTING) and DENV2 NS2B3 using serial deletion hSTING constructs. (A) Schematic of hSTING C-terminal cytoplasmic domain serial deletion constructs. aa, amino acid; ER, endoplasmic reticulum. (B) DENV2 NS2B3 can cleave all hSTING C-terminal cytoplasmic domain serial deletion constructs. Western blots of total cell lysates collected at 24 h post-cotransfection of various hSTING-FLAG constructs with empty vector (EV), DENV2 NS2B3-HA, mutNS2B3-HA, or ZIKV NS2B3-HA. (C) DENV2 NS2B3 can be pulled down by all hSTING C-terminal cytoplasmic domain serial deletion constructs. Anti-FLAG immunoprecipitation was performed on total cell lysates collected at 24 h post-cotransfection of various hSTING-FLAG constructs with empty vector, DENV2 NS2B3-HA, mutNS2B3-HA, or ZIKV NS2B3-HA. (D) Schematic of hSTING N-terminal cytoplasmic domain and cytoplasmic loop domain deletion constructs. (E) DENV2 NS2B3 protease cannot cleave hSTING cytoplasmic loop deletion constructs. Western blots of total cell lysates collected at 24 h post-cotransfection of hSTING N-terminal deletion constructs with empty vector and DENV2 NS2B3-HA. (F) DENV2 NS2B3 can be pulled down by all human STING (hSTING) N-terminal cytoplasmic domain and cytoplasmic loop domain deletion constructs. Anti-FLAG immunoprecipitation was performed on total cell lysates collected at 24 h post-cotransfection of various hSTING-FLAG constructs with empty vector and DENV2 NS2B3-HA. (G) Schematic of proposed DENV NS2B3 and hSTING interaction.

In our effort to shed more light on the mechanism of STING antagonism by the DENV protease complex, we decided to identify regions on hSTING that interact with NS2B3 by cloning hSTING serial deletion constructs and performing coexpression and immunoprecipitation assays with the protease. We believe this mapping strategy can help us identify NS2B3 contact sites on hSTING that may serve as important binding and cleaving determinants for cleavage to occur, in addition to the proposed RG motif. The first three constructs are all C-terminal-tail deletion mutants: hSTINGΔ339 lacks the C-terminal domain that is important for TANK binding kinase 1 (TBK1) activation, the deletion in hSTINGΔ160 further deletes the cGAMP binding domain, and the deletion in hSTINGΔ136 ablates the dimerization domain, leaving only the transmembrane (TM) domain (Fig. 2A).

We first coexpressed our hSTING constructs with DENV2 NS2B3 in HEK293T cells. We observed cleaving of all of the hSTING constructs when coexpressed with DENV2 NS2B3 and, again, a lack of cleaving by ZIKV NS2B3 (Fig. 2B). This suggests that the C-terminal tail may not contain important determinants for the binding and cleavage of hSTING. Then, we performed immunoprecipitation (IP) with the same constructs using anti-FLAG tag agarose beads and noted that NS2B3 could be pulled down with just the N-terminal transmembrane domain of hSTING (Fig. 2C). In addition, we observed that ZIKV NS2B3 could also be pulled down using our wild-type (WT) hSTING construct. It is possible that during ZIKV infection, a different form of antagonism occurs that prevents STING signaling without cleaving.

Next, we carried out serial deletions in the N terminus of hSTING by constructing three more recombinant proteins: hSTINGΔ21 lacks the N-terminal tail, hSTINGΔ68-78 has half of the cytoplasmic loop domain deleted, and hSTINGΔ79-91 has the other half of the loop deleted instead (Fig. 2D). When subjecting the constructs to overexpression together with DENV2 NS2B3 in HEK293T cells, we found that the N-terminal tail region is not needed for hSTING cleavage. Nonetheless, when either loop was missing, we did not observe any cleavage with our constructs (Fig. 2E). This result agrees with previous reports showing the R78/G79 sequence situated on the cytoplasmic loop to be an important cleavage determinant. We then performed an immunoprecipitation assay to check whether any of our deletions disrupted binding between hSTING and the protease complex (Fig. 2F). Surprisingly, NS2B3 was pulled down by all of our constructs. We hypothesized that either (i) NS2B3 straddles the top of the loop and thus can interact with either side of the hSTING cytoplasmic loop or (ii) NS2B3 interacts with hSTING through protein-protein interactions within the endoplasmic reticulum (ER) membrane (Fig. 2G). We then tested our hypotheses by investigating the interactions of individual components of DENV2 NS2B3 with hSTING.

### NS3 associates with the C terminus of hSTING, whereas NS2B binds to the transmembrane domain.

To get a deeper understanding of the dynamic between hSTING and DENV2 protease protein-protein interactions, we set out to separate the viral protease subunits and test them individually when transfecting them together with different hSTING constructs into HEK293T cells.

**FIG 3:**
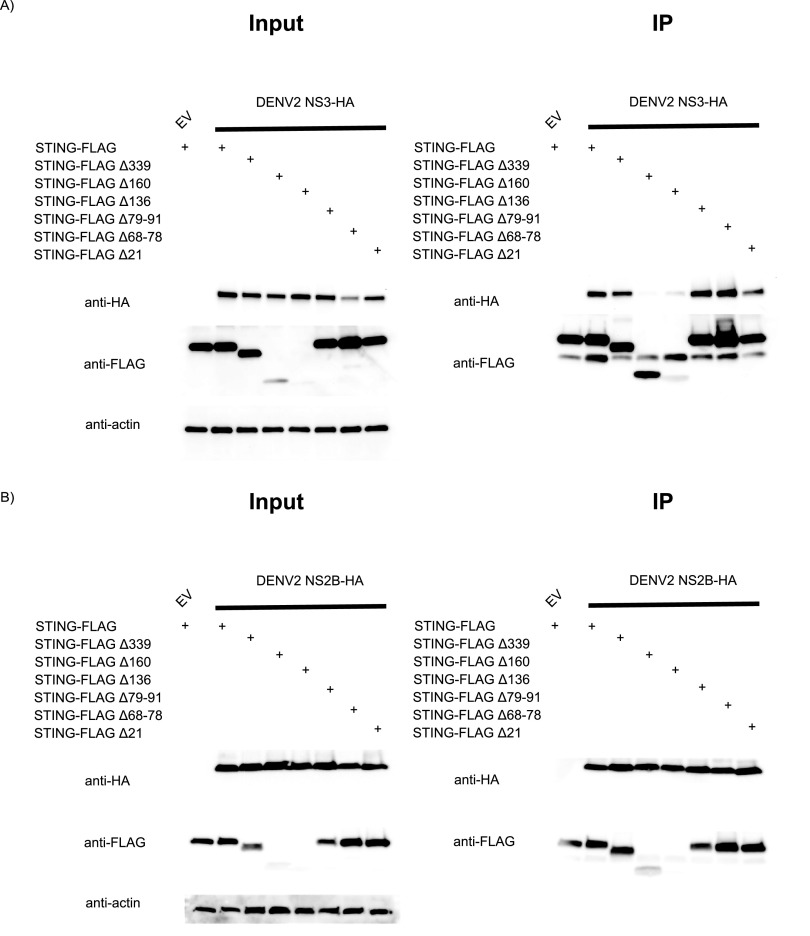

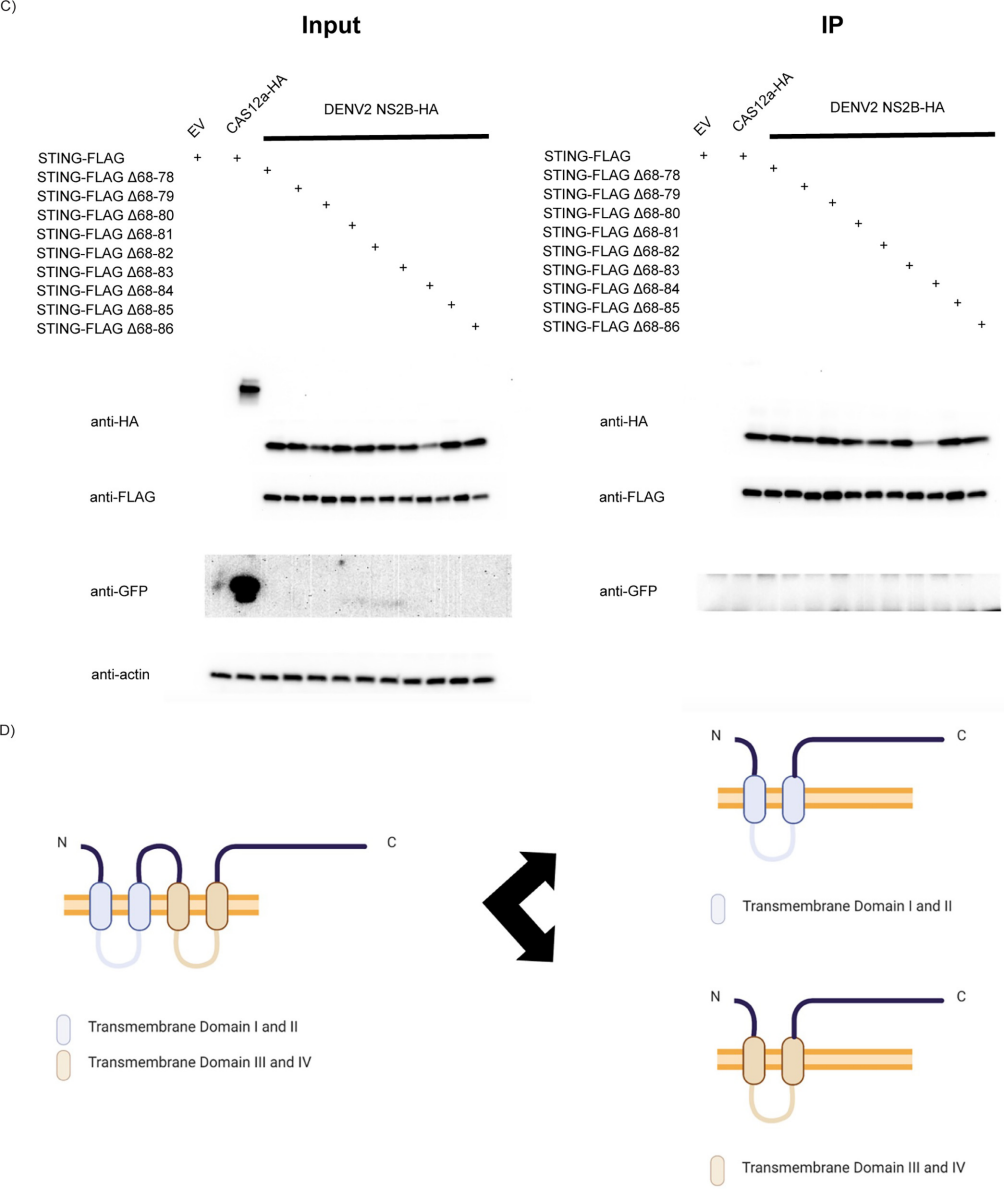

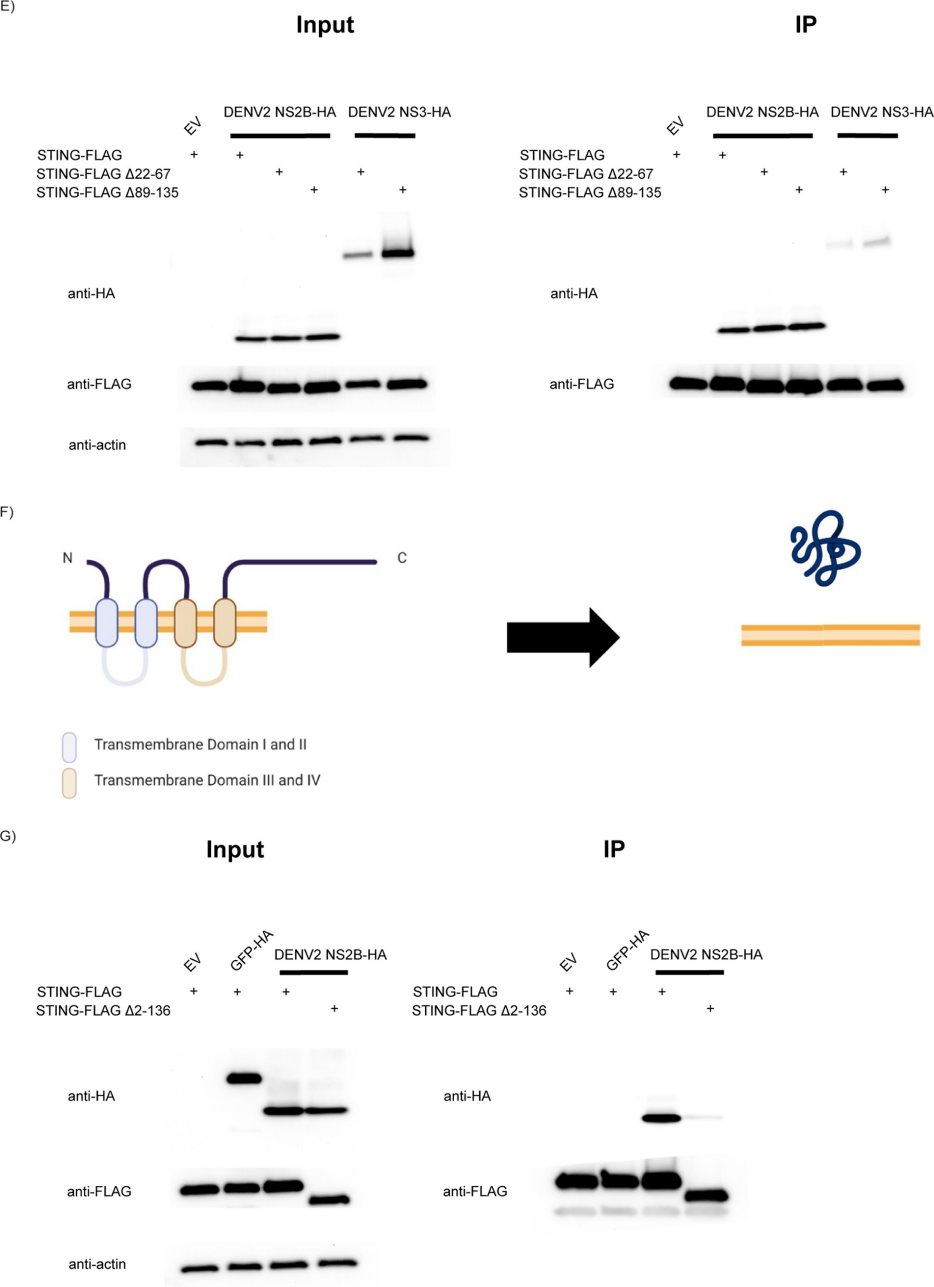
Mapping protein-protein interaction between hSTING and DENV2 NS2B and NS3 individually using serial deletion hSTING constructs. (A) DENV2 NS3 associates with the C-terminal cytoplasmic domain of hSTING. Anti-FLAG immunoprecipitation was performed on total cell lysates collected at 24 h post-cotransfection of various hSTING-FLAG constructs with empty vector and DENV2 NS3-HA. (B) hSTING N-terminal and C-terminal cytoplasmic domains are not required for NS2B association. Anti-FLAG immunoprecipitation was performed on total cell lysates collected at 24 h post-cotransfection of various hSTING-FLAG constructs with empty vector and DENV2 NS2B-HA. (C) hSTING cytoplasmic loop domain is not required for NS2B association. Anti-FLAG immunoprecipitation was performed on total cell lysates collected at 24 h post-cotransfection of hSTING-FLAG cytoplasmic loop serial deletion constructs with empty vector, CAS12a-HA, a DNA endonuclease control, or DENV2 NS2B-HA. (D) Schematic of two transmembrane domain deletion constructs of hSTING. (E) Partial deletion of hSTING transmembrane domains disrupts DENV2 NS3, but not NS2B, association. Anti-FLAG immunoprecipitation was performed on total cell lysates collected at 24 h post-cotransfection of two different hSTING-FLAG transmembrane domain deletion constructs with empty vector, DENV2 NS2B-HA, or NS3-HA. (F) Schematic of the complete transmembrane domain deletion construct of hSTING. (G) Complete deletion of hSTING transmembrane domains disrupts DENV2 NS2B association. Anti-FLAG immunoprecipitation was performed on total cell lysates collected at 24 h post-cotransfection of hSTING-FLAG complete transmembrane domain deletion construct with empty vector, GFP-HA, or DENV2 NS2B-HA.

First, we transfected NS3 alone along with all the hSTING serial deletion constructs into HEK293T cells and performed IP with anti-FLAG tag agarose beads (Fig. 3A). We were not surprised to see a lack of cleavage for all of our constructs in the input, as NS3 alone does not show catalytic activity. However, we identified a novel interaction between DENV2 NS3 and the cGAMP binding domain of hSTING, and not the N-terminal tail region. When hSTINGΔ160 and hSTINGΔ136 (expressed at low levels even when large amounts of plasmids were used for transfection) were coexpressed with NS3, we could not pull down NS3. This suggests that NS3 may exhibit a stand-alone anti-STING function, in addition to its proteolytic role as a viral protease. This was already evidenced in our previous publication, where NS3 alone was shown to counteract the cGAS/STING pathway in a IFN-β reporter assay (12). This was noteworthy because NS3 is the serine protease, and if it does not bind to the hSTING cytoplasmic loop directly where the putative cleaving site is likely to be, then either the cleavage site is not on the cytoplasmic loop or NS2B functions as a bridge between the loop and NS3. Therefore, we further characterized the ability of NS2B alone to interact with our hSTING constructs to test the hypotheses described above.

After transfecting the protease subunit NS2B alone with the hSTING serial deletion constructs and subjecting the samples to immunoprecipitation pulldowns, we noticed no cleavage of hSTING constructs and that NS2B could be pulled down with hSTING lacking the C-terminal tail, and also with one lacking the N-terminal tail (Fig. 3B). Given that NS2B’s known function is to interact with and activate the catalytic triad of NS3 and that it has multiple transmembrane domains, we hypothesize that NS2B may interact with the transmembrane domain of hSTING in order to guide NS3, which binds elsewhere on hSTING, closer to the cleaving site on the cytoplasmic loop between transmembrane domain II (TMII) and TMIII (Fig. 2G). To test our hypothesis, we first made serial deletions in the cytoplasmic loop of hSTING and cotransfected NS2B with the hSTING constructs, and then we performed anti-FLAG IP to rule out the possibility that NS2B straddles the cytoplasmic loop and therefore can be pulled down when either side is missing (Fig. 3C). We noticed that NS2B could consistently be pulled down by constructs of hSTING harboring progressively shorter cytoplasmic loops, to the point where the loop has been deleted completely. This, combined with earlier observations using tail deletion constructs, suggests that NS2B may interact mainly with the TM domains of hSTING instead. To assess this possibility, we designed two more hSTING constructs in which two of the four TM domains were selectively deleted (Fig. 3D). We coexpressed the hSTING TM deletion constructs with both NS2B and NS3 and performed IP (Fig. 3E). The result suggests that the incomplete disruption of the TM domains of hSTING is not sufficient to abolish the protein-protein interactions between NS2B and hSTING, even when this can destabilize the binding of NS3 to hSTING. Finally, an hSTING^C-term^ construct was made in which the TM domains are completely absent (Fig. 3F). When IP was performed, we saw a significant reduction in interaction between NS2B and hSTING^C-term^ (Fig. 3G).

Taken together, our observations using hSTING serial deletion constructs support our hypothesis that NS2B3 interaction domains on hSTING are important determinants for its cleavage, since NS2B serves not only as a cofactor in activating the NS3 serine protease but also as a chaperon in bringing NS3, which binds elsewhere on hSTING, closer to the cleavage site(s).

### hmSTING chimeras with human cytoplasmic loop can be cleaved by NS2B3.

**FIG 4:**
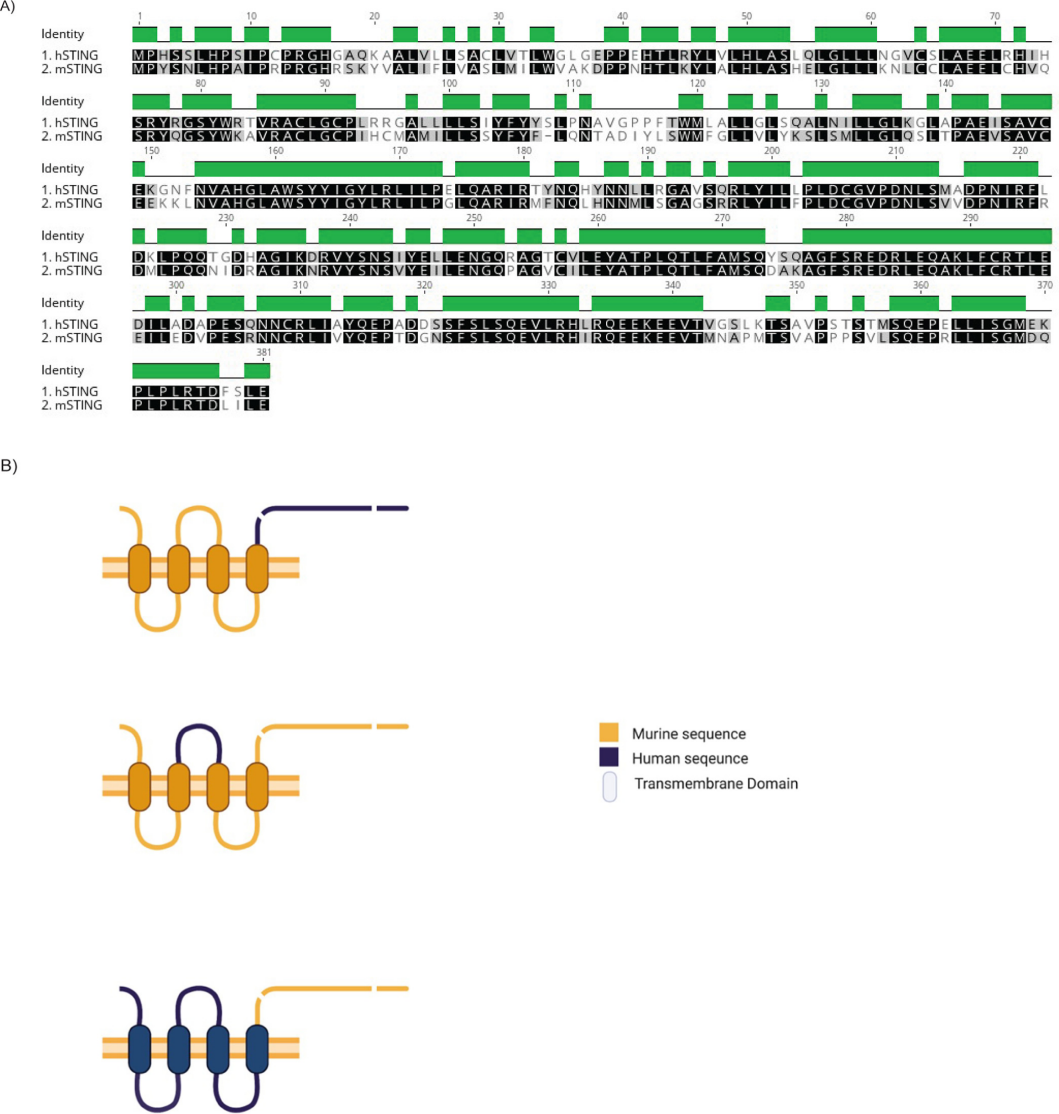

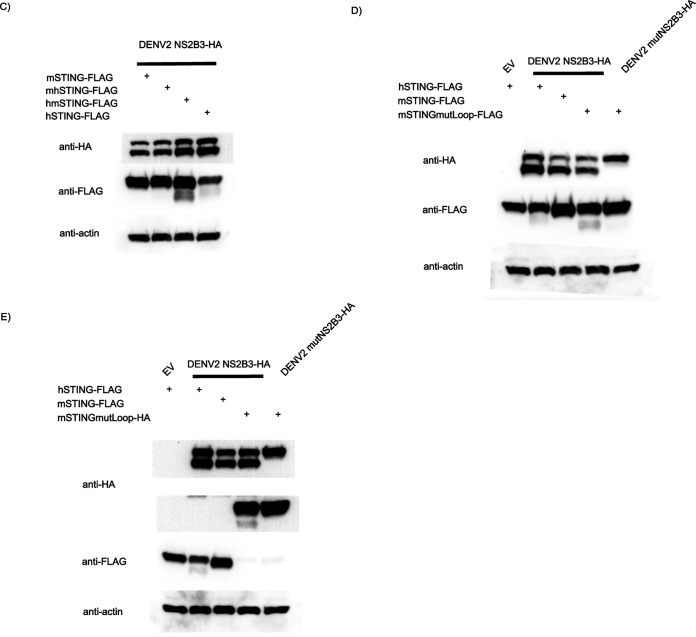

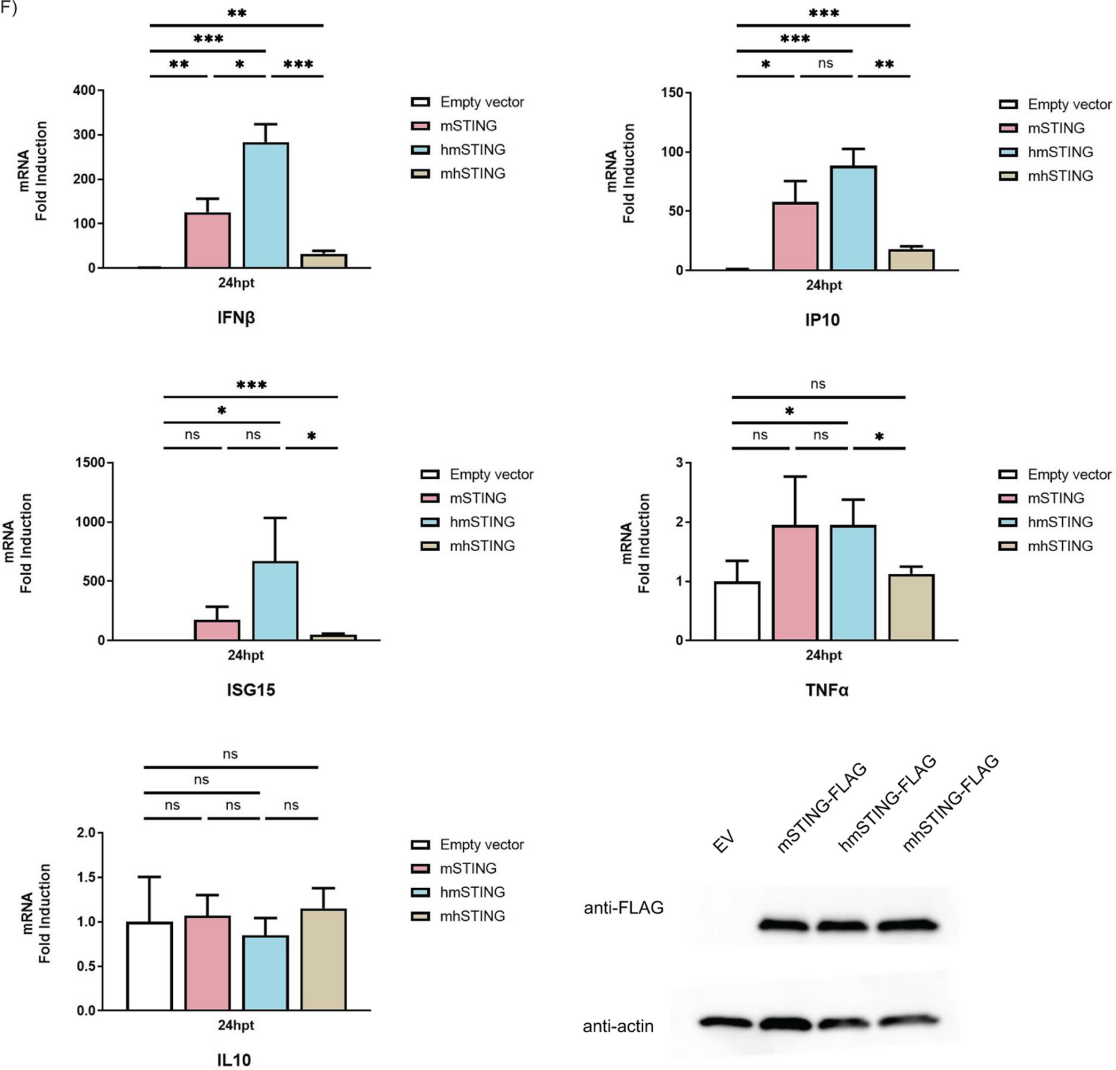

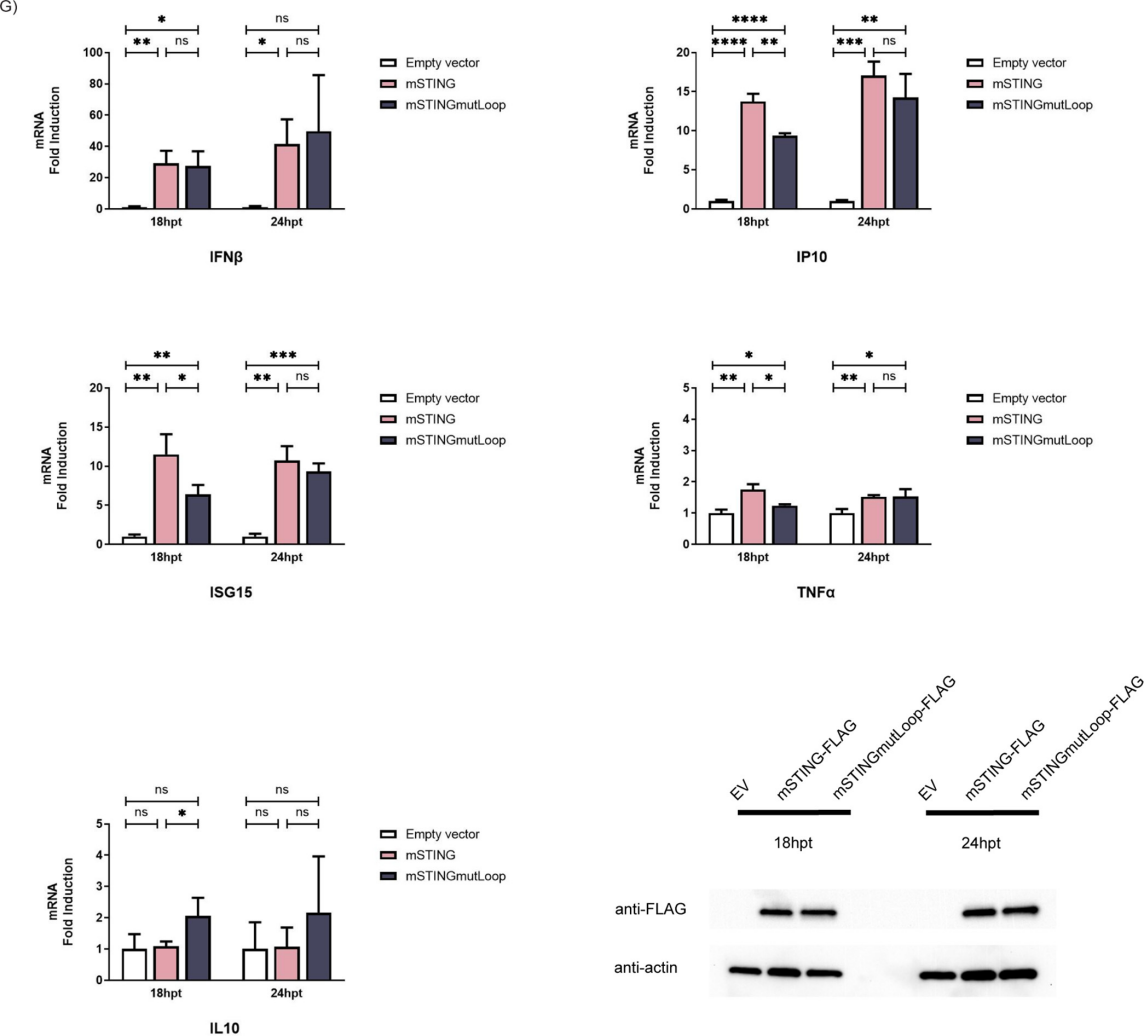
Characterization of human-mouse STING chimeras. (A) Pairwise global alignment of human and murine STING using Geneious Alignment algorithm (Geneious Prime 2021.2.2). Identical sequences are highlighted in black. Green bars indicate identical sequence positions. Similar sequences based on Blosum62 score matrix (threshold = 1) are highlighted in gray, and dissimilar sequences are not highlighted. (B) Schematic of the chimeric STING constructs. (C) DENV2 NS2B3 can cleave the hmSTING chimeric construct. Western blots of total cell lysates collected at 24 h post-cotransfection of STING chimeric constructs with DENV2 NS2B3-HA. (D) DENV2 NS2B3 can cleave the mSTINGmutLoop construct. Western blots of total cell lysates collected at 24 h post-cotransfection of mSTINGmutLoop construct with DENV2 NS2B3-HA and mutNS2B3-HA. (E) DENV2 NS2B3 can cleave the mSTINGmutLoop construct with a different tag. Western blot of total cell lysates collected at 24 h post-cotransfection of mSTINGmutLoop construct of another tag with DENV2 NS2B3-HA and mutNS2B3-HA. (F) mRNA levels of IFN-β, ISG15, IP10, TNF-α, and IL-10 assessed by RT-qPCR after empty vector, WT mSTING, and human-mouse STING chimera constructs were transfected into STING-KO murine macrophage RAW 264.7 cells after 24 h. Results are expressed as relative *C_T_* values against murine housekeeping gene *TUBA1A* (mean value ± standard deviation [SD]) from three biological replicates for all conditions. *, *P* ≤ 0.05; **, *P* ≤ 0.01; ***, *P* ≤ 0.001; ns, nonsignificant. Plasmid expression levels were assessed by Western blotting. (G) mRNA levels of IFN-β, ISG15, IP10, TNF-α, and IL-10 assessed by RT-qPCR after empty vector, WT mSTING, and human-mouse STING chimera construct were transfected into STING-KO murine macrophage RAW 264.7 cells after 24 h and 48 h. Results are expressed as relative *C_T_* values against murine housekeeping gene *TUBA1A* (mean value ± SD) from three biological replicates for all conditions. *, *P* ≤ 0.05; **, *P* ≤ 0.01; ***, *P* ≤ 0.001; ns, nonsignificant. Plasmid expression levels were assessed by Western blotting.

As discussed above, a mouse model to study DENV infection that is both immunocompetent and able to support robust viremia to recapitulate many of the clinical signs of disease we observe in the field is lacking. In the STING field specifically, many attempts have been made to try to swap a stretch of human STING that has a putative DENV protease cleaving site into the murine STING to render it cleavable (Fig. 4A). So far, only Stabell et al. have demonstrated a sign of success by mutating glutamine at position 78 to arginine on mSTING (17). However, under the longer exposure that was required to see the cleaved product in a Western blot, it would seem the WT mSTING could also be cleaved by DENV2 NS2B3 in that experiment.

To design a STING that can not only be efficiently cleaved by NS2B3 protease but can also signal properly in murine cells, we designed three different human-murine STING chimeric constructs: hmSTING contains a human N-terminal transmembrane domain and a murine C-terminal tail, mhSTING harbors a murine N-terminal transmembrane domain and a human C-terminal tail, and mSTINGmutLoop is minimally mutated from mSTING to include only the stretch of the human cytoplasmic loop domain (Fig. 4B).

First, we coexpressed all three constructs with DENV2 NS2B3 in HEK293T cells to determine their susceptibility to cleavage by NS2B3 (Fig. 4C and D). It would appear that hmSTING is easily cleavable compared to mhSTING and mSTING (Fig. 4C). We also observed cleavage of mSTINGmutLoop by NS2B3 and a tentative smaller cleavage product compared to that of hSTING (Fig. 4D). We proposed that since NS3 interacts with the C-terminal tail and NS2B with the TM domain of hSTING, a change to murine-specific amino acid sequences in these regions of this chimeric STING might affect the location of the cleaving site(s) through a different protein complex arrangement. The catalytically inactive version of NS2B3 was used as a negative control, and another version of mSTINGmutLoop with a hemagglutinin (HA) tag was used to confirm our observations (Fig. 4E).

The relative abilities of the mutant STING constructs to induce an IFN-I promoter were tested in murine cells. As has been previously reported, the transfection of STING alone can induce its own activation and the subsequent activation of its downstream effectors (such as IFN regulatory factor 3 [IRF3] and IFN-I) (13, 17, 23). After transfecting our human-mouse chimeric STING proteins into murine macrophage RAW 264.7 cells that are STING-KO, we collected RNA at two different time points and assessed the mRNA levels of key proinflammatory markers (Fig. 4F and G). We observed significant increases in IFN-I, IP10, and ISG15 transcript levels for both mSTING and hmSTING, especially at 24 h posttransfection (Fig. 4F). There was also an increase in the IFN-I induction level measured by real-time quantitative PCR (RT-qPCR) for mhSTING compared to the induction for the empty vector control, although to a lesser degree than for the WT mSTING. This may be due to the facts that the C-terminal tail of STING has important domains for downstream signaling and that a human C-terminal tail would not be as efficient in IFN-I activation as a murine one in a murine cellular environment. We also assessed the transcript levels for tumor necrosis factor alpha (TNF-α) and interleukin 10 (IL-10) (activators for NF-κB and anti-inflammatory pathways) as additional controls for the pathway specificity of this activation (24, 25). Small increases in TNF-α transcript levels were observed for mSTING and hmSTING at 24 h posttransfection, and no significant changes could be detected in IL-10 levels.

We also found that the IFN-I activation profile of mSTINGmutLoop is similar to that of WT mSTING (Fig. 4G). This suggests that both hmSTING and mSTINGmutLoop can signal efficiently in the type I IFN cascade in mouse cells, making them good candidates for the development of an immunocompetent humanized mouse model of DENV infection. Our next step was to assess whether our chimeric constructs could be cleaved under infection conditions and whether they would exhibit proviral or antiviral effects when expressed in murine cells.

### DENV infectivity of STING-deficient murine cells transfected with human-mouse STING chimeras.

**FIG 5:**
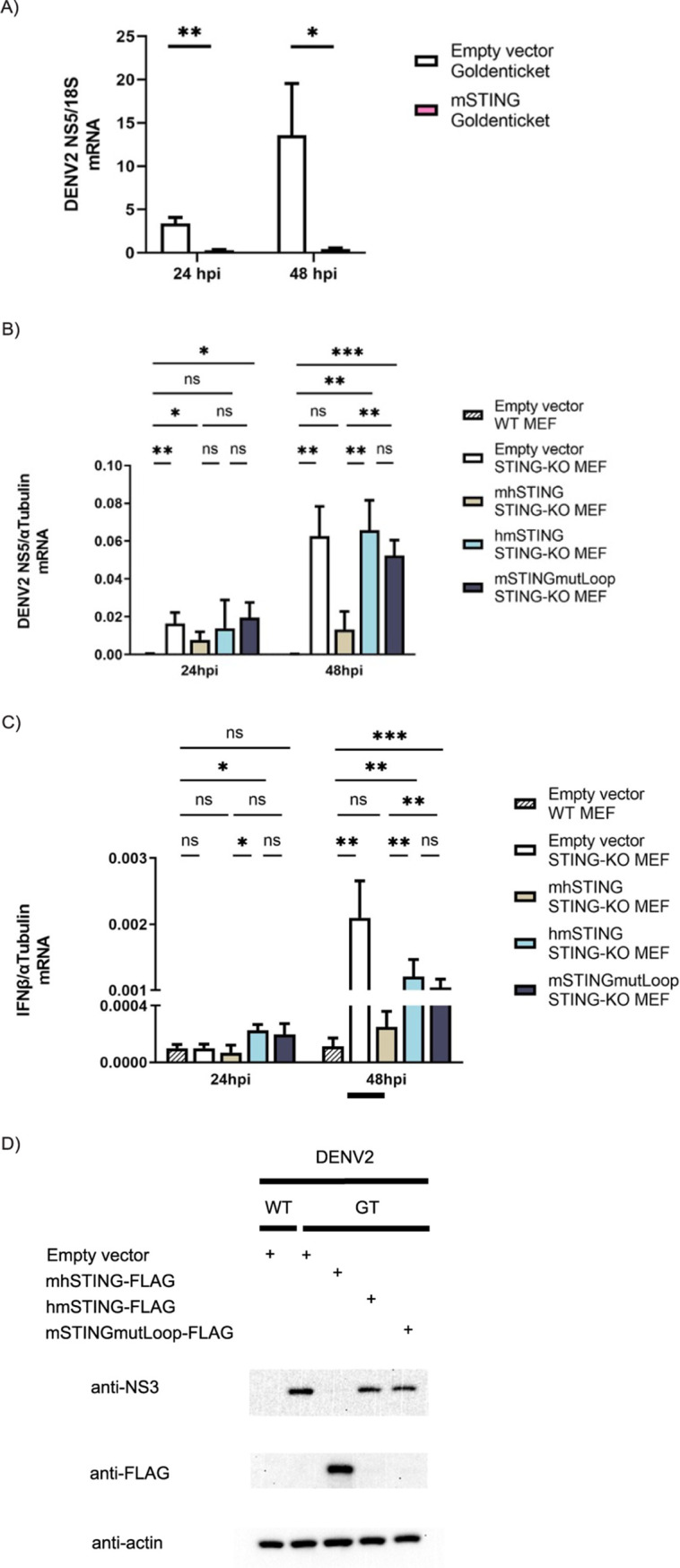
Goldenticket mouse embryonic fibroblast cells expressing hmSTING and mSTINGmutLoop constructs can support DENV2 replication. (A) mSTING restricts DENV2 replication in goldenticket MEFs. mRNA level of DENV2 NS5 was assessed by RT-qPCR after empty vector and mSTING construct were transfected into STING-KO MEFs after 24 h and then infected with DENV2 at an MOI of 1 for another 24 and 48 h. (B) hmSTING and mSTINGmutLoop constructs support DENV2 replication in goldenticket MEFs. (C) DENV2 replication triggers IFN-β response in goldenticket MEFs. mRNA levels of DENV2 NS5 and IFN-β were assessed by RT-qPCR after empty vector and human-mouse chimeric STING constructs were transfected into STING-KO MEFs after 24 h and then infected with DENV2 at an MOI of 1 for another 24 and 48 h. Results are expressed as relative *C_T_* values against housekeeping genes (mean value ± SD) from three biological replicates for all conditions. *, *P* ≤ 0.05; **, *P* ≤ 0.01; ***, *P* ≤ 0.001; ns, nonsignificant. (D) hmSTING and mSTINGmutLoop constructs were degraded during DENV2 infection. Protein expression levels of human-mouse STING chimeras were assessed by Western blotting after transfected WT and goldenticket (GT) MEFs were infected with DENV2 at an MOI of 1 for 48 h.

To test whether WT mSTING can inhibit DENV replication in goldenticket mouse embryonic fibroblast (MEF) cells that are STING deficient, we infected them with DENV2 at an MOI of 1 after transfecting them with mSTING for 24 h. We then collected the RNA and assessed the level of active viral replication using RT-qPCR (Fig. 5A). We found that mSTING expression can inhibit DENV2 replication in Goldenticket MEFs to a significant degree.

Next, we transfected goldenticket MEFs with our human-mouse chimeric constructs for 24 h and then infected them with DENV2 at an MOI of 1 (Fig. 5B). After infection, RNA was collected at two different time points, and levels of viral replication were assessed. To serve as a reference, we also included WT MEF cells that have an intact cGAS/STING pathway in the transfection-then-infection experiment, and we detected viral infection among WT MEFs, albeit at very low levels. We observed that STING-KO cells, as well as STING-KO MEFs expressing the hmSTING construct, saw an increase in DENV replication levels over time. At 48 h postinfection, the hmSTING condition supported much higher DENV2 replication levels than WT MEFs. Not surprisingly, the mhSTING condition did not seem to support a robust DENV2 replication past 24 h postinfection.

mSTINGmutLoop was also examined under the infection experimental condition, and it was observed to support a higher level of DENV replication than the WT MEFs at both time points (Fig. 5B). There was also no difference between hmSTING and mSTINGmutLoop in their abilities to support DENV replication in STING-KO MEFs at both time points.

Furthermore, we assessed antiviral responses in the STING-KO MEFs by measuring IFN-β transcript levels by RT-qPCR in additional experiments (Fig. 5C). In general, we observed that the levels of IFN-β mRNA in response to viral infection were consistent between experiments. For the experimental conditions allowing high DENV replication (STING-KO cells transfected with empty vector, hmSTING, and mSTINGmutLoop plasmids) at a later time point, high levels of IFN-β responses were detected. With the WT MEFs and STING-KO MEFs expressing the uncleavable mhSTING, which restricts viral replication, their IFN-β induction levels were significantly lower. This suggests that an active antiviral response is generated in STING-KO murine cells, likely carried out by other important innate immune players (such as members of the RNA-sensing pathway), once DENV overcomes the initial barrier of infection.

Finally, the expression levels of all human-mouse chimeras were assessed using Western blotting under the same experimental setup (Fig. 5D). We noticed robust degradation of hmSTING and mSTINGmutLoop under DENV2 infection conditions compared to the degradation of mhSTING. Furthermore, the NS3 protein levels correlate with the viral mRNA levels assessed by RT-qPCR, as shown by the results in Fig. 5B.

## DISCUSSION

One major challenge in the DENV research field is the lack of animal models that can exhibit clinical manifestations similar to those seen during human infection (16). In particular, for mice and other rodents, DENV does not replicate to high levels and manifest in disease (21). It is believed that this rodent tropism barrier for DENV infection was the result of the evolutionary pressure exerted on the virus to evade the human immune response and not the rodents’ (14–16). This problem can sometimes be overcome by using immunocompromised mice or mice expressing human immune elements. However, useful as they are in offering a different insight into DENV pathogenesis, none of the mouse models we have can fully recapitulate the important aspects of dengue disease in humans. Therefore, the goal of our current study is to leverage what is known about the interactions between NS2B3 and hSTING and design a human-mouse STING construct that can (i) be stably expressed and function normally in murine cells under mock conditions and (ii) be efficiently cleaved by NS2B3 protease, thus allowing DENV replication in those cells. This is the first step in developing a transgenic mouse model with a functioning innate immune system that can simultaneously be targeted by DENV for antagonism (14).

Our laboratory has previously demonstrated that a catalytically active DENV2 NS2B3 can decrease IFN-I production in human dendritic cells, the primary targets for DENV infection *in vivo* (26). In the decade that followed this discovery, multiple attempts were made to characterize the protein-protein interaction between STING and NS2B3 of DENV (13, 14, 17, 18, 27, 28). Of the two potential sites that have been proposed, the evidence lately has been increasingly pointing toward the arginine-78 and glycine-79 motif on hSTING. While there are still outstanding issues to be resolved before we can be certain, our observations give credence to this hypothesis (Fig. 2C and E).

In addition to identifying one or more short sequences of amino acids on STING where the cleavage by NS2B3 is likely to occur, it is also valuable to characterize any binding domains on these two proteins because, other than direct antagonization, we know nothing about their protein-protein interaction. We discovered that even when the cytoplasmic loop where cleaving of STING is likely to occur is disrupted, DENV2 NS2B3 can still associate with the protein, possibly through the interaction with the C-terminal tail (Fig. 2G). And we verified our hypothesis by performing IP with just the NS3 unit of the protease and hSTING serial deletion constructs (Fig. 3A). Interestingly, it appears that NS3’s binding to the cGAMP binding domains of hSTING functions independently of its proteolytic activity. This perhaps can help explain how NS3 alone can function as a cGAS/STING pathway antagonist (12). Moreover, we observed that NS2B associates with the TM domains of hSTING (Fig. 3E and G).

We hypothesized that NS2B serves as the bridge between NS3 and hSTING, and thus, its binding to our chimeric construct is important for their effective cleavage. Towards that goal, we designed and constructed two recombinant proteins that maintained a mouse C-terminal tail but with different portions of human N-terminal sequences: hmSTING and mSTINGmutLoop (Fig. 4B). We aimed to strike a balance between optimal mSTING functioning and maximal NS2B3 cleavage; therefore, their difference is that while hmSTING has a complete N-terminal human sequence, mSTINGmutLoop has only the 20 human amino acids that make up the cytoplasmic loop. As a control, we also made mhSTING, in which mSTING is replaced with an hSTING C-terminal tail.

When tested for their ability to induce IFN-I activation, hmSTING and mSTINGmutLoop behaved similarly to WT mSTING, while mhSTING in general was less potent (Fig. 4F and G). This suggests that the C-terminal tail is important for mSTING activation, as it is in hSTING. After confirming that the constructs could effectively induce IFN-I during activation, we tested them for their ability to control DENV2 replication when expressed in murine cells (Fig. 5B). In these experiments, we included WT MEFs with a functioning murine innate immune system as controls. When infected with DENV at an MOI of 1, WT MEFs show lower DENV replication levels inside the cells than MEFs that are STING deficient. The difference is more significant at 48 h postinfection. This suggests that having a functioning mSTING is necessary for controlling DENV replication in MEFs. Under an MOI of 1, we observed robust DENV replication levels under hmSTING transfection conditions at 48 h postinfection compared to the replication level in WT MEFs. The mSTINGmutLoop condition also demonstrated a higher level of DENV replication than WT MEFs at both time points (Fig. 5B). In addition, we also observed higher IFN-β induction levels for the cells that supported DENV replication (Fig. 5C). We hypothesize that even if the murine cells we tested are missing STING, a critical component of the DNA-sensing pathway, active DENV replication can still trigger cellular immune responses through other pathways, possibly by the innate immune RNA-sensing mechanism. Even so, it appears that it is sufficient to simply remove STING in murine cells for DENV to establish a productive replication cycle. Finally, both hmSTING and mSTINGmutLoop constructs are degraded during DENV2 infection compared to mhSTING (Fig. 5D). This pattern of almost complete degradation of STING under the DENV2 infection condition was also observed for endogenous STING in human monocyte-derived dendritic cells 48 h postinfection (14).

Together, the evidence so far suggests that our hmSTING and mSTINGmutLoop human-mouse chimeric constructs can function normally in murine cells when activated and be antagonized by DENV during infection. We hypothesize that this is due to the preservation of the C-terminal mSTING signaling domain in these constructs while harboring an N-terminal human region for optimal viral NS2B3 interaction. As this study has proven the efficacies of our two candidate constructs in an overexpression system, our next step moving forward will be to express them in mice and develop a mouse model of DENV that is immunocompetent and can support a higher level of DENV replication at the same time.

Thus, in this study, we have determined the specific amino acids involved in the interaction between STING and the DENV NS2B3 protease complex that enable the protease to efficiently cleave STING to facilitate viral replication. Using our data, we were able to design chimeric versions of murine and human STING that allow efficient cleavage by the DENV protease complex while preserving the innate immune functions of STING in murine cells.

## MATERIALS AND METHODS

### Cell lines.

Human embryonic kidney-293T (HEK-293T) cells were purchased from ATCC (catalog number CRL-3216) and maintained in Dulbecco’s modified Eagle’s medium with 5% fetal bovine serum, 100 U/mL l-glutamine, and 100 U/mL penicillin/streptomycin. C6/36 cells from Aedes albopictus mosquitoes were originally received from J. Munoz-Jordan (CDC, Puerto-Rico) and maintained in RPMI medium with 10% fetal bovine serum at 33°C. Baby hamster kidney cells (BHK) (gift from S. Shresta, La Jolla Immunology Institute) were maintained in Glasgow minimal essential medium (GMEM) with 10% fetal bovine serum and 20 mM HEPES. Murine RAW 264.7 cells were purchased from InvivoGen and maintained according to the recommended handling procedures provided by the manufacturer (WT, catalog code rawl-isg, and STING KO, catalog code rawl-kostg). MEFs, both WT and goldenticket, were gifts from Jonathan Miner (WUSTL) and were maintained in Dulbecco’s modified Eagle’s medium (DMEM) with 20% fetal bovine serum. In addition, all tissue culture reagents were purchased from Invitrogen. All cell lines were tested in our laboratory for the presence of mycoplasma using the MycoAlert mycoplasma detection kit from Lonza (catalog number LT07-118) and were mycoplasma free.

### Viruses.

All virus preparations were tested in our laboratory for the presence of mycoplasma using the MycoAlert mycoplasma detection kit from Lonza (catalog number LT07-118) and were mycoplasma free. Dengue virus serotype 2 (DENV2) strain 16681 was used for infection studies. DENV2 was grown in C6/36 mosquito cells for 6 days, as our group described previously (12, 14). Briefly, C6/36 cells were infected by DENV2, and cell supernatants were collected, clarified, and stored at −80°C. Virus stocks of DENV3 strain PR-6, DENV4 strain H-241, YFV strain 17D, and ZIKV strain French Polynesia 2013 were used to generate the respective NS2B3 constructs. The titers of viral preparations were determined by limiting-dilution plaque assay on BHK cells, as our group described previously (12, 14, 22).

### Generation of NS2B3 constructs.

Our group has previously described the generation and characterization of DENV2 NS2B3, NS2B, and NS3 plasmids (12, 14, 26). RNAs from DENV3, DENV4, YFV, and ZIKV viral preparations were extracted using TRIzol from Invitrogen (catalog number 15596026) according to the manufacturer’s protocol. The RNA concentration was determined via spectrophotometer at 260 nm and then stored at −80°C. The RT reaction was performed with the iScript cDNA synthesis kit (catalog number 1708891; Bio-Rad) using random hexamer priming according to the manufacturer’s instructions. PCR was then performed with CloneAmp HiFi PCR premix from TaKaRa Bio (catalog number NC0384799) according to the manufacturer’s protocol to generate NS2B3, NS2B, or NS3 inserts, and the products cloned into the pGA13 mammalian expression vector using a combination of In-Fusion cloning and site-directed mutagenesis methods. EcoRI and BamHI restriction sites were used for cloning with the In-Fusion HD cloning kit from Clontech (catalog number 638920). The Q5 site-directed mutagenesis kit from New England Biolabs (catalog number E0554S) was also used. Eton Bioscience synthesized relevant primers for cloning, and all plasmids were sent for sequencing by Eton Bioscience and verified before being purified using Qiagen plasmid kits (catalog number 27115).

### Generation of STING constructs.

Both human and murine STING plasmids have been previously generated and characterized by our group (14). Serial deletions of human STING functional regions and the construction of human-mouse STING chimeras were generated and cloned into the pGA13 mammalian expression vector using a combination of In-Fusion cloning and site-directed mutagenesis methods as mentioned before.

### Infection of WT and goldenticket MEFs.

WT and goldenticket MEFs were seeded in 12-well or 96-well plates and transfected with the respective plasmids. Twenty-four hours after transfection, cells were infected with DENV2 at an MOI of 1. Infections were allowed to proceed for 1 h in serum-free DMEM before the infection medium was removed, and cells were maintained with regular growth medium up to the respective time points. At the selected time points, RNA and protein lysates were collected and then stored at −80°C. All experiments were performed at least twice.

### Cellular lysate preparation.

Cellular lysates were prepared by incubating cells in radioimmunoprecipitation assay (RIPA) lysis buffer (catalog number 89900; Thermo Scientific) supplemented with EASYpack protease inhibitor cocktail mini, EDTA-free, cOmplete ultra tablets (catalog number 05892791001; Roche) for 5 min at 4°C. Then, all lysates were subjected to one overnight −80°C freeze-thaw cycle. Quantification of protein levels was performed with the colorimetric Bradford assay (catalog number 5000006; Bio-Rad), using bovine serum albumin (BSA) to generate a standard curve.

### Immunoblot analysis.

Cellular lysates were resuspended, in a 4:1 ratio, in 4× NuPAGE LDS sample buffer (catalog number NP0007; Invitrogen) supplemented with 2-mercaptoethanol and heated at 70°C for 10 min in a heating block (Fisher Scientific). All samples were then loaded on Mini-Protean TGX precast gels (catalog numbers 4561034, 4561044, 4561084, and 4561094; Bio-Rad), and the denatured proteins were separated by electrophoresis. Protein was then wet transferred to polyvinylidene difluoride (PVDF) membranes (catalog number 1620177; Bio-Rad). Blots were blocked with Tris-buffered saline (TBS) supplemented with 0.1% Tween 20 with 5% milk for 1 h at room temperature, followed by 4°C overnight incubation with primary antibodies. The primary antibodies used included anti-FLAG antibody (catalog number F7425; Millipore), anti-HA antibody (catalog number H3663; Sigma-Aldrich), and anti-β-actin antibody (catalog number A2228; Sigma-Aldrich), all at 1:5,000 dilution, and anti-green fluorescent protein (GFP) antibody (catalog number GF28R; Invitrogen) at 1:1,000 dilution. The secondary antibodies used were anti-mouse antibody (catalog number NA931V; GE Healthcare) and anti-rabbit antibody (catalog number NA934V; GE Healthcare) at 1:5,000 dilution. The bands were detected using the SuperSignal chemiluminescence system (catalog number 34579; Thermo Scientific).

### Immunoprecipitation assay.

Immunoprecipitation was carried out using the EZview Red Anti-FLAG M2 affinity gel (catalog number F2426; Sigma-Aldrich). The affinity gel was washed 3 times in cold RIPA lysis buffer (mentioned above). Then, whole-cell lysates with the same level of protein abundance were mixed with the affinity gel and incubated on a rotator for 1 h at 4°C. After incubation, the affinity gel was spun down and washed 4 times for 10 min each in cold RIPA lysis buffer. After the last wash, the affinity gel was resuspended in 4× NuPAGE LDS sample buffer (catalog number NP0007; Invitrogen) supplemented with 2-mercaptoethanol and heated at 70°C for 10 min in a heating block (Fisher Scientific). Samples were then stored at −20°C until immunoblot analysis.

### Phylogenetic tree construction and amino acid sequence alignment.

Phylogenetic tree construction using the amino acid sequences of NS2B3 of DENV (GenBank accession numbers EU848545.1, NC_001474.2, NC_001475.2, and NC_002640.1), YFV (GenBank accession number MG051217.1), and ZIKV (GenBank accession number AHZ13508.1) and pairwise global alignment of hSTING (GenBank accession number MF622062.1) and mSTING (GenBank accession number MF622063.1) were all performed on the Geneious Prime 2021.2.2 platform.

### RAW 264.7 cell RNA isolation and RT-qPCR.

RNA from RAW 264.7 cells was extracted using the *Quick*-RNA miniprep kit (catalog number R1054; Zymo Research) following the manufacturer’s protocol, including the in-column DNase treatment. The RNA concentration was determined via spectrophotometer at 260 nm and then stored at −80°C until the next step. The RT reaction was performed with the iScript cDNA synthesis kit (catalog number 1708891; Bio-Rad) using random hexamer priming according to the manufacturer’s instructions. Evaluation of the relative expression levels of murine cytokines was performed using iQ SYBR green supermix (catalog number 1708880; Bio-Rad) according to the manufacturer’s instructions. Real-time PCR analysis was performed on the Bio-Rad 1000C thermal cycler. The PCR temperature profile was 95°C for 10 min, followed by 40 cycles of 95°C for 10 s and then 60°C for 60 s. Expression levels were calculated based on the mean cycle threshold (*C_T_*) values from three technical replicates using the murine housekeeping gene *TUBA1A* to normalize the data.

### WT and goldenticket MEF RNA isolation and one-step RT-qPCR.

MEFs were lysed in 200 μL of TRIzol and placed on ice for 30 min. RNA extraction was done via Direct-zol-96 RNA kits (catalog number R2054; Zymo Research) according to the manufacturer’s protocol. The concentration of RNA was quantified via a spectrophotometer at 260 nm. One-Step RT-qPCR was performed using the Luna universal one-step RT-qPCR kit (catalog number E3005S; New England Biolabs) using 2 μL of total RNA per condition on the Bio-Rad 1000C thermal cycler. The PCR temperature profile was 55°C for 20 min and then 95°C for 5 min, followed by 40 cycles of 95°C for 10 s and then 60°C for 60 s. Expression levels were calculated based on the mean *C_T_* values from three technical replicates, using the murine housekeeping gene *TUBA1A* or 18S small subunit rRNA to normalize the data.

### Schematics.

All schematics used in the article were prepared with Biorender.com.

### Statistical analysis.

The unpaired, two-tailed Student’s *t* test was used for direct comparisons. Specific analyses used are listed in the figure legends, as well as different *P* value cutoffs. Significances were reported when the *P* value was <0.05. No data point was excluded when analyzing these data.

10.1128/msphere.00914-21.1 FIG S1 Infectivity of DENV2 versus DENV4 in human foreskin fibroblasts (hFFs) assessed by plaque assays. The titers were determined by limiting-dilution plaque assay on BHK cells after supernatants from infected hFFs were collected at the respective time points. Error bars represent standard deviations calculated from the results from three technical replicates. Download FIG S1, TIF file, 0.7 MB. Copyright © 2022 Zhu et al. 2022 Zhu et al. https://creativecommons.org/licenses/by/4.0/ This content is distributed under the terms of the Creative Commons Attribution 4.0 International license.

## References

[1] Oliveira ERA, Gonçalves AJS, Costa SM, Azevedo AS, Mantuano-Barradas M, Nogueira ACMA, Alves AMB. 2016. Aspects of T cell-mediated immunity induced in mice by a DNA vaccine based on the dengue-NS1 antigen after challenge by the intracerebral route. PLoS One 11:e0163240. doi:10.1371/journal.pone.0163240. 27631083 PMC5024998

[2] Tang WW, Grewal R, Shresta S. 2015. Influence of antibodies and T cells on dengue disease outcome: insights from interferon receptor-deficient mouse models. Curr Opin Virol 13:61–66. doi:10.1016/j.coviro.2015.04.007. 26001278

[3] Bhatt S, Gething PW, Brady OJ, Messina JP, Farlow AW, Moyes CL, Drake JM, Brownstein JS, Hoen AG, Sankoh O, Myers MF, George DB, Jaenisch T, Wint GRW, Simmons CP, Scott TW, Farrar JJ, Hay SI. 2013. The global distribution and burden of dengue. Nature 496:504–507. doi:10.1038/nature12060. 23563266 PMC3651993

[4] Tuiskunen Bäck A, Lundkvist Å. 2013. Dengue viruses: an overview. Infect Ecol Epidemiol 3:19839. doi:10.3402/iee.v3i0.19839. PMC3759171 24003364

[5] Rothman AL. 2010. Cellular immunology of sequential dengue virus infection and its role in disease pathogenesis. Curr Top Microbiol Immunol 338:83–98. doi:10.1007/978-3-642-02215-9_7. 19802580

[6] Malavige GN, Huang L-C, Salimi M, Gomes L, Jayaratne SD, Ogg GS. 2012. Cellular and cytokine correlates of severe dengue infection. PLoS One 7:e50387. doi:10.1371/journal.pone.0050387. 23209731 PMC3510251

[7] Guzman MG, Alvarez M, Halstead SB. 2013. Secondary infection as a risk factor for dengue hemorrhagic fever/dengue shock syndrome: an historical perspective and role of antibody-dependent enhancement of infection. Arch Virol 158:1445–1459. doi:10.1007/s00705-013-1645-3. 23471635

[8] Wilder-Smith A, Ooi E-E, Horstick O, Wills B. 2019. Dengue. Lancet 393:350–363. doi:10.1016/S0140-6736(18)32560-1. 30696575

[9] Zhu T, Fernandez-Sesma A. 2020. Innate immune DNA sensing of flaviviruses. Viruses 12:979. doi:10.3390/v12090979. PMC7552040 32899347

[10] Webb LG, Veloz J, Pintado-Silva J, Zhu T, Rangel MV, Mutetwa T, Zhang L, Bernal-Rubio D, Figueroa D, Carrau L, Fenutria R, Potla U, Reid SP, Yount JS, Stapleford KA, Aguirre S, Fernandez-Sesma A. 2020. Chikungunya virus antagonizes cGAS-STING mediated type-I interferon responses by degrading cGAS. PLoS Pathog 16:e1008999. doi:10.1371/journal.ppat.1008999. 33057424 PMC7591055

[11] Zheng Y, Liu Q, Wu Y, Ma L, Zhang Z, Liu T, Jin S, She Y, Li Y‐P, Cui J. 2018. Zika virus elicits inflammation to evade antiviral response by cleaving cGAS via NS1-caspase-1 axis. EMBO J 37:e99347. doi:10.15252/embj.201899347. 30065070 PMC6138430

[12] Aguirre S, Luthra P, Sanchez-Aparicio MT, Maestre AM, Patel J, Lamothe F, Fredericks AC, Tripathi S, Zhu T, Pintado-Silva J, Webb LG, Bernal-Rubio D, Solovyov A, Greenbaum B, Simon V, Basler CF, Mulder LCF, García-Sastre A, Fernandez-Sesma A. 2017. Dengue virus NS2B protein targets cGAS for degradation and prevents mitochondrial DNA sensing during infection. Nat Microbiol 2:17037. doi:10.1038/nmicrobiol.2017.37. 28346446 PMC7457382

[13] Yu C-Y, Chang T-H, Liang J-J, Chiang R-L, Lee Y-L, Liao C-L, Lin Y-L. 2012. Dengue virus targets the adaptor protein MITA to subvert host innate immunity. PLoS Pathog 8:e1002780. doi:10.1371/journal.ppat.1002780. 22761576 PMC3386177

[14] Aguirre S, Maestre AM, Pagni S, Patel JR, Savage T, Gutman D, Maringer K, Bernal-Rubio D, Shabman RS, Simon V, Rodriguez-Madoz JR, Mulder LCF, Barber GN, Fernandez-Sesma A. 2012. DENV inhibits type I IFN production in infected cells by cleaving human STING. PLoS Pathog 8:e1002934. doi:10.1371/journal.ppat.1002934. 23055924 PMC3464218

[15] Ashour J, Morrison J, Laurent-Rolle M, Belicha-Villanueva A, Plumlee CR, Bernal-Rubio D, Williams KL, Harris E, Fernandez-Sesma A, Schindler C, García-Sastre A. 2010. Mouse STAT2 restricts early dengue virus replication. Cell Host Microbe 8:410–421. doi:10.1016/j.chom.2010.10.007. 21075352 PMC3310429

[16] Chen RE, Diamond MS. 2020. Dengue mouse models for evaluating pathogenesis and countermeasures. Curr Opin Virol 43:50–58. doi:10.1016/j.coviro.2020.09.001. 32950933 PMC7774505

[17] Stabell AC, Meyerson NR, Gullberg RC, Gilchrist AR, Webb KJ, Old WM, Perera R, Sawyer SL. 2018. Dengue viruses cleave STING in humans but not in nonhuman primates, their presumed natural reservoir. Elife 7:e31919. doi:10.7554/eLife.31919. 29557779 PMC5860865

[18] Ding Q, Gaska JM, Douam F, Wei L, Kim D, Balev M, Heller B, Ploss A. 2018. Species-specific disruption of STING-dependent antiviral cellular defenses by the Zika virus NS2B3 protease. Proc Natl Acad Sci USA 115:E6310–E6318. doi:10.1073/pnas.1803406115. 29915078 PMC6142274

[19] Shiryaev SA, Kozlov IA, Ratnikov BI, Smith JW, Lebl M, Strongin AY. 2007. Cleavage preference distinguishes the two-component NS2B-NS3 serine proteinases of dengue and West Nile viruses. Biochem J 401:743–752. doi:10.1042/BJ20061136. 17067286 PMC1770841

[20] Simmons JS, St John JH, Reynolds FH. 1931. Experimental studies of dengue. Philipp J Sci 44:1–251.

[21] Bente DA, Rico-Hesse R. 2006. Models of dengue virus infection. Drug Discov Today Dis Models 3:97–103. doi:10.1016/j.ddmod.2006.03.014. 18087566 PMC1949394

[22] Hamlin RE, Rahman A, Pak TR, Maringer K, Mena I, Bernal-Rubio D, Potla U, Maestre AM, Fredericks AC, Amir E-AD, Kasarskis A, Ramos I, Merad M, Fernandez-Sesma A. 2017. High-dimensional CyTOF analysis of dengue virus-infected human DCs reveals distinct viral signatures. JCI Insight 2:e92424. doi:10.1172/jci.insight.92424. PMC5499363 28679950

[23] Ishikawa H, Barber GN. 2008. STING is an endoplasmic reticulum adaptor that facilitates innate immune signalling. Nature 455:674–678. doi:10.1038/nature07317. 18724357 PMC2804933

[24] Bouwmeester T, Bauch A, Ruffner H, Angrand P-O, Bergamini G, Croughton K, Cruciat C, Eberhard D, Gagneur J, Ghidelli S, Hopf C, Huhse B, Mangano R, Michon A-M, Schirle M, Schlegl J, Schwab M, Stein MA, Bauer A, Casari G, Drewes G, Gavin A-C, Jackson DB, Joberty G, Neubauer G, Rick J, Kuster B, Superti-Furga G. 2004. A physical and functional map of the human TNF-alpha/NF-kappa B signal transduction pathway. Nat Cell Biol 6:97–105. doi:10.1038/ncb1086. 14743216

[25] Iyer SS, Cheng G. 2012. Role of interleukin 10 transcriptional regulation in inflammation and autoimmune disease. Crit Rev Immunol 32:23–63. doi:10.1615/critrevimmunol.v32.i1.30. 22428854 PMC3410706

[26] Rodriguez-Madoz JR, Belicha-Villanueva A, Bernal-Rubio D, Ashour J, Ayllon J, Fernandez-Sesma A. 2010. Inhibition of the type I interferon response in human dendritic cells by dengue virus infection requires a catalytically active NS2B3 complex. J Virol 84:9760–9774. doi:10.1128/JVI.01051-10. 20660196 PMC2937777

[27] Dalrymple NA, Cimica V, Mackow ER. 2015. Dengue virus NS proteins inhibit RIG-I/MAVS signaling by blocking TBK1/IRF3 phosphorylation: dengue virus serotype 1 NS4A is a unique interferon-regulating virulence determinant. mBio 6:e00553-15. doi:10.1128/mBio.00553-15. 25968648 PMC4436066

[28] Su C-I, Kao Y-T, Chang C-C, Chang Y, Ho T-S, Sun HS, Lin Y-L, Lai MMC, Liu Y-H, Yu C-Y. 2020. DNA-induced 2′3′-cGAMP enhances haplotype-specific human STING cleavage by dengue protease. Proc Natl Acad Sci USA 117:15947–15954. doi:10.1073/pnas.1922243117. 32576686 PMC7354927

