# Supplementary figures and images for: Generation and Characterization of Human-Mouse STING Chimeras That Allow DENV Replication in Mouse Cells
Source: mSphere. 2022 Apr 28;7(3):e00914-21. doi: 10.1128/msphere.00914-21 (PMC9241525; doi:10.1128/msphere.00914-21)

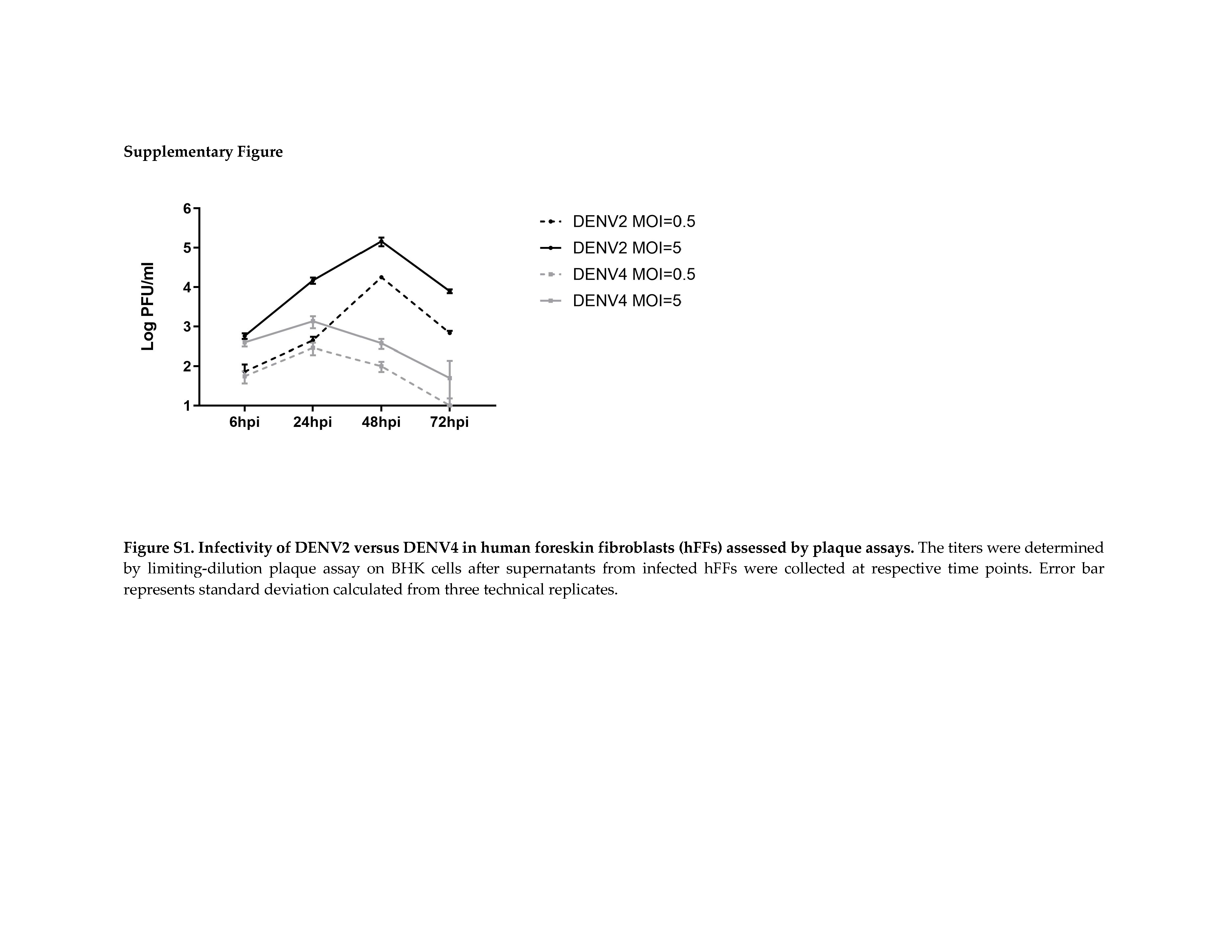

Supplement: FIG S1 [file msphere.00914-21-s0001.tif]
